# Adult rat ultrasonic vocalizations and reward: Effects of propranolol and repeated cocaine administration

**DOI:** 10.1177/02698811241268894

**Published:** 2024-08-12

**Authors:** Leyla Erden, Adithi Sundarakrishnan, Paul BS Clarke

**Affiliations:** Department of Pharmacology and Therapeutics, McGill University, Montreal, QC, Canada

**Keywords:** Ultrasonic vocalizations, propranolol, cocaine, amphetamine, rats, sensitization, adrenergic, positive affect, chronic

## Abstract

**Background:**

Mechanisms underlying psychostimulant euphoria remain poorly understood. In adult rats, positive emotional states are associated with alterations in 50-kHz ultrasonic vocalizations (USVs): specifically, “trill” calls are promoted over “flat” calls. Here, we investigated the effects of acute and repeated cocaine administration, and—based on previous findings with amphetamine—their possible dependence on beta-adrenergic receptors.

**Methods:**

Adult male Long-Evans rats received intraperitoneal drug or saline injections before daily USV recording. Fourteen 50-kHz call subtypes were analyzed. In Experiments 1 and 2, cocaine (1–10 mg/kg) and propranolol (10 mg/kg) were tested alone. In Experiment 3, propranolol/cocaine interactions were sought within a conditioned place preference (CPP) procedure. Experiment 4 investigated acute and chronic cocaine effects (Phase 1), and propranolol/cocaine interactions either in an open field (Phase 2) or within a CPP procedure (Phase 3).

**Results:**

In drug-naïve animals, cocaine increased the 50-kHz call rate, with sensitization developing rapidly. After more extended exposure, cocaine now also increased the relative prevalence of trill versus flat calls; effects on other subtypes were also revealed. The beta-blocker propranolol prevented neither cocaine CPP nor cocaine effects on USV emission or locomotion but exerted significant USV-related effects when given alone. CPP magnitude and USV-related measures were uncorrelated.

**Conclusions:**

With long-term intraperitoneal administration, cocaine can alter the relative prevalence of several 50-kHz call subtypes; its ability to promote trill versus flat calls, in particular, is consistent with a positive affect interpretation. Cocaine’s behavioral effects (i.e., USV-related, locomotor, CPP) appear independent of beta-adrenergic receptor activity.

## Introduction

Although psychostimulants induce well-known rewarding effects in human subjects, the underlying neurochemical mechanisms have been only partially identified. In an early study, amphetamine euphoria was greatly attenuated or abolished by alpha-methyl-p-tyrosine, a drug that inhibits the synthesis of catecholamine neurotransmitters (Jonsson et al., 1969). Subsequent studies have focused largely on dopamine (DA), but while it contributes to reinforcing effects of psychostimulants, its contribution to drug-induced euphoria is not well supported (Berridge and Kringelbach, 2015; Leyton et al., 2007; Nutt et al., 2015). In adult rats, cocaine can produce rewarding effects through different mechanisms, depending on the route of administration; specifically, conditioned place preference (CPP) resulting from intravenous (IV) cocaine is reported to be DA-dependent, whereas CPP following intraperitoneal cocaine appears DA-*independent* (Sellings et al., 2006; Spyraki et al., 1987).

Cocaine and amphetamine enhance noradrenergic as well as dopaminergic neurotransmission (Florin et al., 1994; Tanda et al., 1997), and noradrenergic contributions to psychostimulant reward have been identified using conventional preclinical measures (Schmidt and Weinshenker, 2014). Although most of the relevant studies concern amphetamine rather than cocaine, IV cocaine self-administration was reduced by the beta-blocker propranolol in both rhesus monkeys (Goldberg and Gonzales, 1976) and rats (Harris, 1996). Whether propranolol can also inhibit the acquisition of cocaine CPP appears unreported.

If psychostimulant euphoria is not driven by dopamine, could noradrenaline play a role? To our knowledge, only three preliminary studies have explored this question in human subjects, using subjective effects as the measured outcome. First, propranolol did not significantly reduce amphetamine euphoria, but the antagonist was given only in low oral doses (Jonsson, 1972). In the second, subjective effects of amphetamine were inferred only from the subjects’ observable behavior, and again it is not clear whether propranolol (0.1 mg/kg IV) was administered in a sufficiently high dose (Nurnberger et al., 1984). In the third study, the mixed adrenergic receptor antagonist carvedilol did not detectably blunt the cocaine “high”, but the drug treatment order was not fully randomized (Sofuoglu et al., 2000).

Rodent ultrasonic vocalizations (USVs) are widely used as surrogate measures of emotional state in preclinical models (Brudzynski, 2021; Schwarting, 2023). Rewarding contexts generally increase the overall 50-kHz call rate (Brudzynski, 2021), with frequency-modulated calls predominating (Burgdorf et al., 2011). However, adult rat 50-kHz calls are highly heterogeneous, with 14 main call subtypes identified to date (Wright et al., 2010). Two particular 50-kHz call subtypes, that is, flat and trill calls, appear inversely related across a variety of affective states. Specifically, trill calls predominated during social interactions (Burke et al., 2021; Wright et al., 2010) and after administration of euphorigenic drugs (Best et al., 2017; Wright et al., 2010, 2012a, 2012b), whereas flat calls were more prevalent in negative states associated with D1 DA antagonist or naloxone administration or during morphine withdrawal (Lin et al., 2018; Wright et al., 2013). On this basis, we have proposed that flat and trill calls reflect negative and positive emotional states, respectively (Best et al., 2017; Lin et al., 2018; Wright et al., 2010, 2012a, 2012b).

In an earlier study, we reported that the beta-adrenergic blocker propranolol appeared to prevent amphetamine’s ability to promote trill calls at the expense of flat calls (Wright et al., 2012b). Additional tests with other antagonists showed that propranolol’s effects were due to antagonism of CNS β1 and β2 receptors but not 5-HT1A receptors (Wright et al., 2012b). On this basis, we proposed that amphetamine euphoria in humans may be driven by a CNS beta-adrenergic mechanism. However, this conclusion was necessarily tentative, since low call rates made it impossible to reliably determine whether propranolol, when given alone, altered the 50-kHz call profile (i.e., the relative prevalence of call subtypes). This 2012 study also showed that IV cocaine administration produced the same call profile shift as amphetamine, that is, the promotion of trills over flat calls. However, we did not investigate the effects of intraperitoneal (IP) cocaine administration, or whether propranolol could reverse these effects. We also did not study the long-term effects of cocaine, where sensitization or tolerance can potentially occur (Maier et al., 2010; Meyer et al., 2012; Mu et al., 2009; Sohn et al., 2022; Tripi et al., 2017; Williams and Undieh, 2010).

In the present study, therefore, we first asked whether IP cocaine administration would produce the same 50-kHz call profile shift as seen with amphetamine and IV cocaine, that is, an increased prevalence of trills over flat calls. Subsequent experiments included the prototypical β-blocker propranolol, chosen for two reasons. First, in previous work (Wright et al., 2012b), 50-kHz call profiles during amphetamine sessions were dependent on both β1 and β2 receptors, which are both antagonized by propranolol. Second, propranolol is the most widely used β receptor antagonist in preclinical studies, facilitating comparison with the relevant literature. Accordingly, after first testing for USV-altering effects of propranolol when given alone (Experiment 2), we assessed the effects of propranolol and cocaine, given alone and in combination, on USV, locomotion, and acquisition of cocaine CPP (Experiments 3 and 4). In Experiment 4, we also compared the effects of acute cocaine injection in drug-naïve versus cocaine-experienced rats. When assessing drug effects on USV emission, locomotor activity served as a positive behavioral control, to confirm the activity of cocaine and potentially its interaction with propranolol (Harris, 1996).

## Materials and methods

### Overview of experiments

**Figure 1.**
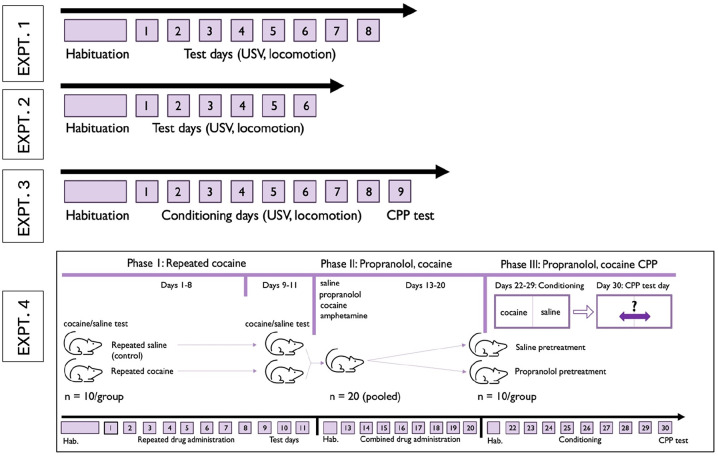
Overview of experiments. Day numbers are shown on each experimental timeline, with only test days numbered.

Four experiments were performed, each with a different batch of rats (Table 1). The timelines for each experiment are shown in Figure 1.

**Table 1.** Overview of experiments.

Expt	Drug(s)	*n*	Apparatus	Dose	USV	CPP	LMA
1	Cocaine dose–response amphetamine	12	Operant chamber	1, 3 and 10 mg/kg, 1 mg/kg	Tested	NT	NT
2	Propranolol alone	12	CPP boxes*	10 mg/kg	Tested	NT	Tested
3	Propranolol + cocaine	24	CPP boxes	10 mg/kg (each drug)	Tested	Tested	Tested
4 phase 1	Acute vs. repeated cocaine	10	CPP boxes*	10 mg/kg	Tested	NT	Tested
4 phase 2	Propranolol + cocaine, amphetamine	20	CPP boxes*	10 mg/kg (each), 1 mg/kg	Tested	NT	Tested
4 phase 3	Propranolol + cocaine	10	CPP boxes	10 mg/kg (each)	Tested	Tested	Tested

* Rats were tested in CPP boxes with bedding rather than drug-paired floor textures.

n: number of rats per condition or group; NT: not tested.

### Subjects

Adult male Long-Evans rats (Charles River Laboratories, Kingston NY, USA) were used, housed two per cage in a humidity- and temperature-controlled colony room. The rats were maintained on a reverse (12 h) light/dark cycle, and food and water were available ad libitum except while testing. Rats were left to acclimate to the colony room for several days upon arrival and were subsequently handled by the experimenter for approximately 5 min each day for 3 days. The number of rats per experiment is shown in Table 1. At the start of behavioral testing, rats weighed as follows (Experiments 1–4, respectively): 214–261, 284–334, 259–303, and 285–383 g. All procedures were approved by the McGill Animal Care Committee in accordance with the guidelines of the Canadian Council on Animal Care.

### Drugs

Cocaine HCl, (R,S)-propranolol HCl, and S(+)-amphetamine sulfate (i.e., d-isomer) were all given by IP injection. Drugs were dissolved in sterile 0.9% saline and administered in a volume of 1 ml/kg body weight. Vehicle control injections were of 0.9% saline. Doses were unless otherwise stated: propranolol HCl 10 mg/kg, cocaine HCl 10 mg/kg, and d-amphetamine sulfate 1 mg/kg. Doses were expressed as salt. Cocaine and amphetamine were given immediately before the 30-min or 60-min test sessions, whereas propranolol was administered 20 min before the 30-min sessions. The standard cocaine dose of 10 mg/kg IP and injection/session timings were based on previous CPP studies (e.g., Nomikos and Spyraki, 1988; Sellings et al., 2006). The doses of propranolol and amphetamine, together with the timing of injections, were also taken from our previous work (Wright et al., 2012b).

### Testing apparatus and data acquisition

In Experiment 1, rats were tested in four sound-attenuated operant conditioning chambers (ENV-007CT, Med Associates, St. Albans, VT, USA), as previously described (e.g., Wright et al., 2010). A condenser ultrasound microphone (CM16/CMPA, Avisoft Bioacoustics, Berlin, Germany) was positioned over a small (5 cm diameter) hole cut in the chamber lid, 15–30 cm from the rat. The two operant levers were kept retracted at all times.

In Experiments 2–4, rats were instead tested in four custom-made CPP boxes (Sundarakrishnan and Clarke, 2022). Each CPP box measured 58 cm × 30 cm × 54 cm (length × width × height), and comprised four vertical walls made of white melamine-coated fiberboard four vertical fiberboard walls, with a rectangular lid made of clear 8-mm-thick Plexiglas™ that was hinged along one long side to allow opening. The four CPP boxes were placed in a quadrant configuration, approximately 2.5 cm apart, on a layer of bedding. Illumination in Experiment 3 was provided by far-red (wavelength > 650 nm) light using a Kodak GBX-2 safelight filter (Vistek, Toronto, Ontario, Canada), whereas in Experiments 2 and 4, infrared (850 nm) LED light sources were used (Tonton Security, ‎Elk Grove Village, IL, USA).

Locomotor activity (LMA) and CPP were both tested in the four boxes described above. In the CPP procedure, two floor textures were used as conditional stimuli: a mesh grid (1 cm^2^ stainless steel wire mesh) and a metal panel containing small holes (4.8 mm diameter, set 6.4 mm apart). Rats do not show a spontaneous preference for either floor texture (T. Scardochio and P. B. S. Clarke, unpublished observation). Square (29 cm × 29 cm) tiles made of either flooring were mounted on melamine frames; two adjacent tiles completely covered the bottom of each CPP cage.

Video recordings were acquired from a camera centered above the four test boxes (see below). In Experiment 3, the video was recorded using a Panasonic model WV-BP330 camera and processed using a Noldus Information Technology video tracking system (EthoVision v 3.0, Leesburg, VA, USA). In Experiments 2 and 4, the camera was a Blackfly S model (FLIR Integrated Imaging Solutions, Richmond, BC, Canada), used with ANY-maze video tracking software (v. 7.15, Stoelting Co., Wood Dale, IL, USA).

In each CPP box, 22-kHz and 50-kHz USVs were recorded with an Avisoft CM16 microphone positioned over an opening (9 cm × 6 cm) cut halfway along one long side of the lid and connected to an UltraSoundGate 416H data acquisition device (Avisoft Bioacoustics). Acoustic recordings were acquired with Avisoft RECORDER v4.4 software, with a 250-kHz sampling rate and 16-bit resolution. To confirm that 50-kHz calls were detectable from all locations within a given test box, but not from adjacent boxes, a custom-made ultrasound emitter was used that generated trains of 50-kHz pulses.

### General testing procedure (all experiments)

On each test day, rats were weighed, transported from the colony to the test room, and then left in their home cages for 15–20 min to acclimatize. Rats were tested four at a time, with a given rat always tested in the same box. At the end of each test day, half of the bedding (Beta Chip^®^, NEPCO, Warrensburg, NY, USA) was replaced, to retain a relatively constant bedding condition across days.

### Details of individual experiments

#### Experiment 1: Cocaine dose–response—USV emission

Each rat (*n* = 12) was tested in eight sessions of 60-min duration, starting immediately after injection of saline (four times), cocaine (1, 3, and 10 mg/kg IP, once each), and amphetamine 1 mg/kg IP (once). The timeline is shown in Figure 1. Drug conditions were ordered according to incomplete Williams square designs.

#### Experiment 2: Propranolol given alone—USV emission and locomotor activity

Each rat (*n* = 12) received two habituation sessions in the test boxes, followed by six once-daily test sessions, comprising three sessions with propranolol (10 mg/kg IP) and three sessions with saline. Sessions occurred on consecutive days (Figure 1), and the drug test order was interleaved across days and counterbalanced across rats (i.e., either SPSPSP or PSPSPS).

#### Experiment 3: Propranolol and cocaine—USV emission and CPP

To test the effects of propranolol on CPP acquisition, a procedure was used consisting of one habituation session, eight drug conditioning sessions, and one final drug-free test session. These sessions occurred across 10 consecutive days (Figure 1). Individual rats were randomly allocated to propranolol or saline pretreatment groups (*n* = 12 per group). For the habituation session, rats were placed in test boxes for 30 min, without injection and with the floor tiles absent. On a given conditioning day, each rat received two injections, spaced 20 min apart. The first injection was of either propranolol 10 mg/kg IP or saline, depending on the group, after which rats were returned to their home cages. The second injection was either cocaine 10 mg/kg IP or saline, depending on the day. Saline and cocaine injections were alternated across days, that is, SCSCSCSC or CSCSCSCS, counterbalanced within each group. After the saline or cocaine injection, the rat was immediately confined to the CPP box for 30 min. Within a conditioning session, the two floor tiles were of the same texture (i.e., both mesh or both holes). A given rat repeatedly received cocaine in combination with one floor texture, and saline challenge in combination with the other floor texture; cocaine-texture pairings were counterbalanced within each pretreatment group. During these conditioning sessions, USVs were recorded and whole-body movements were video-recorded. The test day comprised a single 15-min drug-free session. Each CPP box now contained both floor textures (i.e., two different floor tiles, positioned in a counterbalanced fashion). At the start of the test session, each rat was placed on the midline, straddling the two floor tiles, and the CPP magnitude was then determined.

#### Experiment 4: Propranolol and cocaine—USV emission and CPP

This experiment consisted of three phases. The timeline is shown in Figure 1.

*Experiment 4 Phase 1 (days 1–11)*: Acute cocaine effects before and after repeated cocaine experience. Phase 1 determined the acute effects of cocaine (10 mg/kg) on USV emission in drug-naïve animals (days 1 and 2) and after repeated cocaine exposure (days 9–11). Rats were randomly allocated to two groups (i.e., Repeated Saline or Repeated Cocaine; *n* = 10 per group) and were habituated to the test boxes for 3 days before the 11 consecutive days of testing. On test days 1 and 2, the Repeated Cocaine group was tested once with cocaine and once with saline (*n* = 10/group), in counterbalanced order, whereas the Repeated Saline control group was tested twice with saline. On test days 3–8, the Repeated Cocaine group was tested alternately with saline and cocaine, with the order counterbalanced within the group, while the Repeated Saline group was tested only with saline. On test days 9–11, all 20 rats were tested twice with saline and once with cocaine, the order again being counterbalanced within the group. Sessions lasted 30 min, starting immediately after a cocaine (or saline) injection.

*Experiment 4 Phase 2 (days 12–20) Propranolol pretreatment with cocaine and amphetamine challenge: USV emission and LMA.* Each rat was tested under six different drug conditions, that is all combinations of pretreatment (i.e., saline or propranolol) and acute challenge (i.e., saline, cocaine, or amphetamine). The saline–saline and propranolol–saline tests were administered twice, and hence there were in total eight test sessions per rat. The order of drug testing was counterbalanced according to an incomplete Williams square design; furthermore, on a given day, each drug combination was administered to an equal number of rats from the two Phase 1 groups. Rats received propranolol (or saline) pretreatment, followed 20 min later by a cocaine/amphetamine (or saline) challenge. Sessions lasted 30 min, starting immediately after the cocaine/amphetamine (or saline) injection.

*Experiment 4 Phase 3 (days 21–30) Propranolol pretreatment with cocaine challenge: USV emission and CPP.* The same CPP procedure was used as in Experiment 3. Individual rats were randomly allocated to propranolol (10 mg/kg IP) or saline pretreatment groups (*n* = 10 per group). These groups each comprised five rats previously in the Repeated Saline group, and five rats previously in the Repeated Cocaine group of Phase 1. Rats received propranolol (or saline) pretreatment, followed 20 min later by a cocaine (or saline) challenge. Sessions lasted 30 min, starting immediately after a cocaine (or saline) injection. Exceptionally, on the CPP test day (day 30), rats did not receive any injections and the session lasted 15 min.

### USV analysis

Spectrograms were generated from WAV files by fast Fourier transform (512 points, 75% overlap, FlatTop window, 100% frame size) using Avisoft SASLab Pro (v. 5.2.09) software. Ultrasonic calls were manually selected from spectrograms, and no call intensity threshold was applied. 22-kHz calls (i.e., 20–25 kHz) were treated as a single type, whereas 50-kHz calls (i.e., 25–95 kHz) were categorized (by L.E., blind to drug conditions and group allocation) according to our 14-subtype scheme (Wright et al., 2010), that is, complex, upward ramp, downward ramp, flat, short, split, step-up, step-down, multi-step, trill, flat–trill combination, trill with jumps, inverted-U, and composite. Calls that could not be confidently subtypes were designated as “unclear” or “miscellaneous” (Wright et al., 2010).

Individual rats vary considerably in their 50-kHz call rates, with some rats emitting hundreds of calls per session, especially after cocaine or amphetamine administration. Therefore, to make the call subtyping task manageable, we chose to analyze only a subset of calls in some experiments, as follows. In Experiment 1, the amphetamine test sessions (only) were subjected to 1-in-3 time sampling, that is, retaining for analysis only the middle minute of each successive 3-min block (60–120 s, 240–300 s, etc.). In Experiments 2 and 3, the same 1-in-3 sampling method was applied to all recording sessions, except that low-calling rats that made fewer than ten 50-kHz calls in any drug condition were not time sampled. In Experiment 4, recordings from all phases were either analyzed completely (in low callers, as above) or else time sampled with either a 1-in-5 or 1-in-10 retention ratio (Phases 2 and 3, respectively); to avoid bias, a given rat was subjected to the same time-sampling ratio within each counterbalanced block.

To facilitate time sampling, the original WAV files were submitted to custom-written macros in Audacity^®^ v3.0.2 software (GNU General Public License) such that each session was reduced to 10 equally spaced time slices centered at the mid-point of consecutive 3-min periods. Thus, the 1-in-5 sampling macro extracted ten 36-s slices, and the 1-in-10 sampling macro extracted ten 18-s slices. To be counted, calls had to *start* within a time slice, so to avoid call truncation, each time slice was bordered by additional 1-s “buffer” zones to allow the start and end time of each call to be precisely determined.

As in our previous studies, the primary call subtype measures are reported in terms of relative (i.e., percentage) rather than absolute prevalence. The *relative* prevalence of a given call subtype, unlike the absolute prevalence, is generally not related to the overall 50-kHz call rate (Sundarakrishnan and Clarke, 2022; Wright et al., 2010, 2012b).

### Video analysis

Locomotor activity, which was measured in all Experiments 2–4, was expressed as the total horizontal distance moved per session. CPP magnitude was determined in Experiments 3 and 4, in terms of the time spent on each floor texture. In Experiment 3, the Ethovision software determined the rat to be located on one of the two floor textures at any moment. In Experiment 4, the ANY-Maze software was set so that a rat was identified as being on a given floor texture if 85% of its body profile was superimposed; otherwise, the rat’s position was assigned to a third, neutral zone. For both experiments, the time spent on each texture was then converted to a percentage of time spent on the saline- versus cocaine-paired textures. One rat (rat 3 in Experiment 4) showed clearly erroneous data based on the ANY-Maze tracking, so it was manually re-scored.

### Statistical analysis

Conditions were counterbalanced using Williams square designs (Williams, 1949), as noted above. Statistical software (Systat version 11, SPSS, Chicago, IL, USA) was used for all analyses, and figures were generated using Prism 9 (GraphPad Software, La Jolla, CA, USA). The variables of interest were initially as follows: 50-kHz call rate (i.e., calls/min); the relative (i.e., percentage) prevalence of trill and flat 50-kHz call subtypes; locomotor activity (LMA, i.e., distance moved/30-min session); and CPP magnitude (defined as above). Subsequently, the analysis was expanded to include all 50-kHz call subtypes. Few 22-kHz calls were emitted, and consequently only summary information is provided.

Statistical tests were as follows. Throughout, any single outliers identified using Grubb’s test were excluded from parametric tests (although included in figures). When parametric test assumptions were not met, a nonparametric test was used. Thus, drug conditions or groups were compared using a parametric test (i.e., independent, Welch’s or paired *t*-test) or a nonparametric equivalent (Mann–Whitney U or Wilcoxon signed-rank test, abbreviated as MW and W, respectively). In Experiment 4 Phase 1 days 9–11, two-way ANOVAs were performed, with one between-subject factor (GROUP, i.e., repeated saline vs. repeated cocaine) and one within-subject factor (COCAINE, i.e., acute saline vs. cocaine). In Experiment 4 Phase 2, two-way ANOVAs were performed using the within-subject factors PROPRANOLOL (i.e., saline vs. propranolol) with either COCAINE (i.e., saline vs. cocaine) or AMPHETAMINE (i.e., saline vs. amphetamine). Where ANOVA assumptions were not met, two-way interactions were instead assessed nonparametrically by performing Mann–Whitney U tests on saline-subtracted difference scores. Where a given drug condition was tested in more than one session per subject within the same counterbalanced design, the data were pooled after initial inspection. To test for a CPP, the mean percent time spent on the cocaine-associated side was compared to a neutral value of 50% (one-sample *t*-tests). To compare the CPP magnitude between the propranolol group and the saline control group, an independent *t*-test was used. Possible relations between cocaine’s behavioral effects (e.g., CPP vs. 50-kHz call rate) were assessed using Spearman correlation analysis performed on cocaine-minus-saline difference scores. For all tests, *p* values of 0.01–0.05 were considered a trend, while *p* < 0.01 (two-tailed) was considered statistically significant.

## Results

As in our previous studies, we report all of our call subtype findings in terms of relative prevalence, that is, the contribution of each call subtype as a percentage of all 50-kHz calls emitted in a given drug condition. The absolute prevalence of individual call subtypes (i.e., calls/min) is also reported but not discussed (see Supplemental Table 1).

The main results are summarized in Table 2.

**Table 2.** Summary of results for each drug when tested alone.

Experiment	Drug	Call rate	LMA	FL	TR	CX	FT	SH	UR
1	COC	↑trend	NT	—	↑trend	—	↑trend	↓trend	—
3		↑	↑	↓trend	↑trend	—	—	—	↓
4.1 days 1–2		↑	↑	—	—	—	↑trend	—	—
4.1 days 9–11		↑	↑	—	—	↓	↑	↓trend	—
4.2		↑	↑	—	—	↓	↑trend	—	↓
4.3		↑	↑	↓	↑trend	—	↑	↓	-
2	PROP	—	—	—	—	—	—	—	—
3		↑	—	—	—	—	↑	↓trend	↓
4.2		—	↑	↑	—	—	↑trend	—	—
4.3		—	—	—	—	—	↑trend	—	—
1	AMPH	↑	NT	↓trend	↑	—	↑	↓	—
4.2		↑	↑	—	—	—	↑	—	—

The significance level (i.e., alpha) was set at 1%. Call rate refers to 50-kHz calls only. LMA, locomotor activity. Call subtypes are as follows; FL flat, TR trill, CX complex, FT flat–trill, SH short, and UR upward ramp. Phases 1–3 of Experiment 4 are indicated as 4.1–4.3, respectively. For Experiment 1, only the highest dose (10 mg/kg) is reported, as lower doses did not affect the measures shown.

↑: significant increase; ↓: significant decrease; —: no effect; NT: not tested; ↑trend and ↓trend: *p* = 0.05–0.01; PROP: propranolol; COC: cocaine; AMPH: amphetamine.

### Experiment 1: Cocaine dose–response—USV emission

A total of 22,213 50-kHz calls and 1227 22-kHz calls were identified within the 96 recording sessions (i.e., 8 test days, *n* = 12 rats). In this experiment, only the amphetamine test sessions were time sampled (see Methods). Each rat made at least two 22-kHz calls, but the majority (i.e., 767/1229) of 22-kHz calls were emitted by a single rat. Omitting this rat, 22-kHz calls represented only 2.0% of all calls. Most 22-kHz calls occurred in the four saline sessions. Almost all (i.e., 1222/1227) of the 22-kHz calls lasted less than 100 ms (median duration 32 ms).

**Figure 2.**
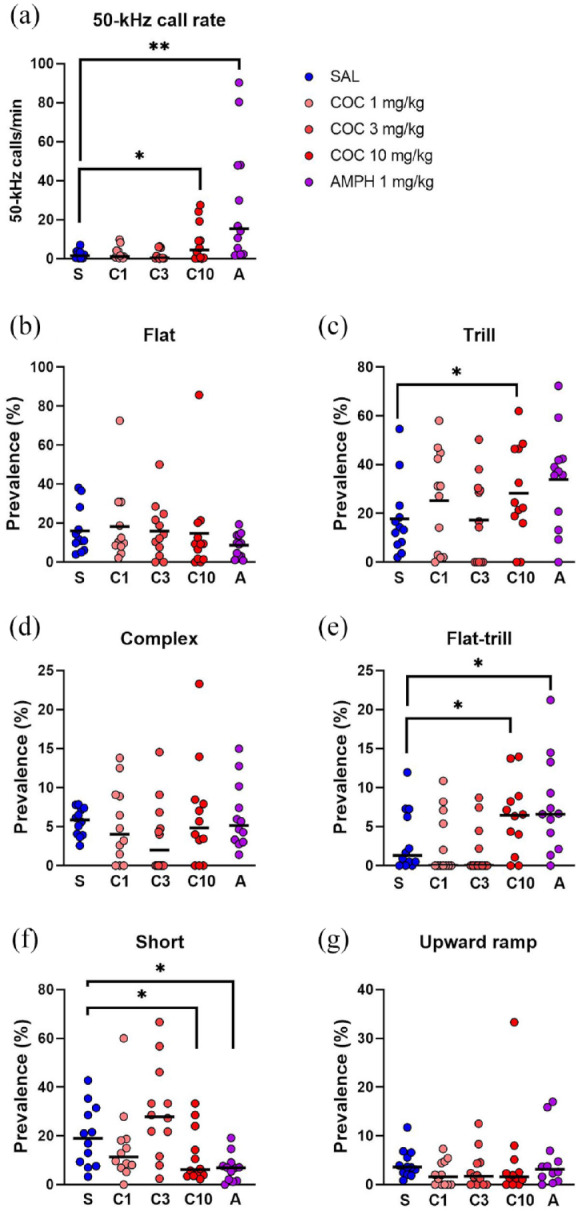
Call rate and selected call subtypes in Experiment 1. *Y*-axes show the 50-kHz call rate (a), the percent prevalence of flat (b), trill (c), and other selected call subtypes (d–g). Each rat was tested in eight sessions: four times with saline, and once each with cocaine 1, 3, 10 mg/kg IP and amphetamine 1 mg/kg, in a counterbalanced design (*n* = 12). This experiment was performed in operant conditioning chambers, whereas all the other experiments were done in the CPP boxes. Each point represents an individual rat. Horizontal lines indicate the median for panels a, and d–g, and the mean for panels b and C. *p < 0.05 (i.e., trend), **p < 0.01, ***p < 0.001 versus saline condition (paired *t*-test or Wilcoxon test).

The 50-kHz call rate was significantly increased by amphetamine (W *p* = 0.0096), with a similar trend for high-dose cocaine (e.g., 10 mg/kg, W *p* = 0.0186; Figure 2(a)). Neither cocaine nor amphetamine significantly altered the percentage of flat or trill calls, although the highest dose of cocaine tended to promote trill calls (*t*_11_ = 2.56, *p* = 0.0263; Figure 2(b) and (c)). Cocaine appeared to affect the relative prevalence of some non-flat/non-trill call subtypes (Supplemental Figure 1(d)–(g)), but only split calls appeared altered by all three doses of cocaine (W *p* = 0.0152, 0.0037, and 0.0109, respectively; Supplemental Figure 1(a)). The highest dose of cocaine also significantly decreased unclear calls (W *p* = 0.0076; Supplemental Figure 1(a)). High-dose cocaine and amphetamine tended to have similar effects on short (Figure 2(f)) and unclear call subtypes (Supplemental Figure 1(a)).

### Experiment 2: Propranolol given alone—USV emission and locomotor activity

**Figure 3.**
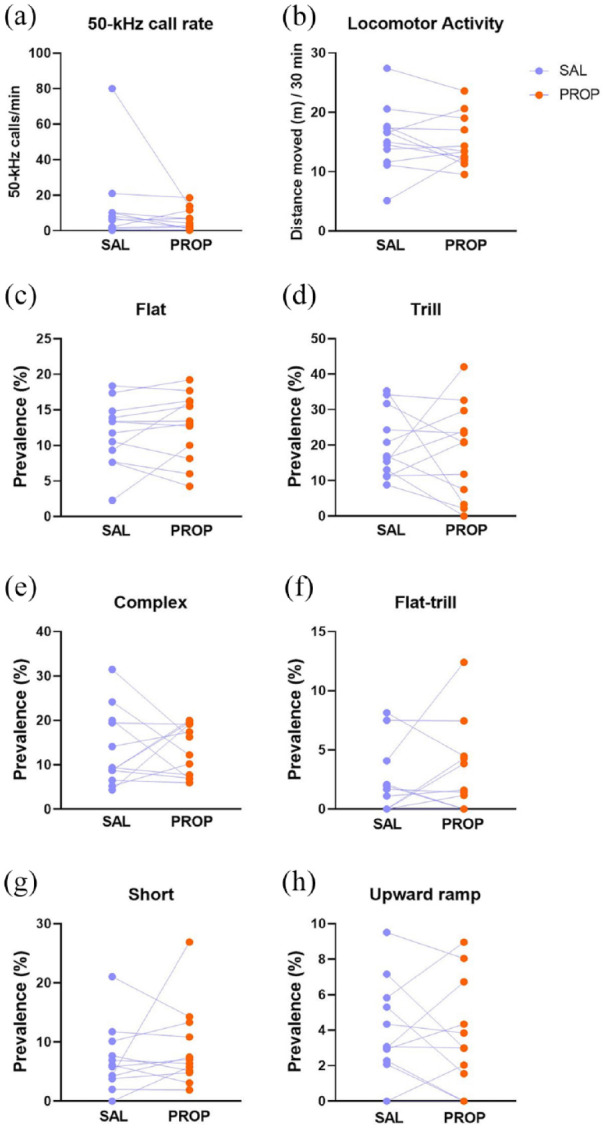
Call rate, locomotor activity, and selected call subtypes in Experiment 2. *Y*-axes show the 50-kHz call rate (a), locomotor activity (b), the percentage prevalence of flat (c), trill (d), and other selected call subtypes (e–h). Rats (*n* = 12) were each tested in a counterbalanced design in which daily tests with propranolol and saline were alternated for 6 days (*n* = 12). Data were collapsed across days within each drug condition. Each line represents an individual rat. *p < 0.05 (i.e., trend), **p < 0.01, ***p < 0.001 versus saline condition (paired *t*-test or Wilcoxon test).

A total of 5,348 50-kHz calls and 10 22-kHz calls were sampled from the 72 sessions (6 days, 3 pairs of saline and propranolol test sessions, *n* = 12 rats). For analysis, after initial inspection data were collapsed within each drug condition. Propranolol did not significantly alter LMA (*t*_11_ = 0.63, *p* = 0.5403; Figure 3(a)) or the 50-kHz call rate (W *p* = 0.2391; Figure 3(b)). Moreover, propranolol did not alter the prevalence of any 50-kHz call subtype (Figure 3(c)–(h)), including flat and trill calls (respectively: *t*_11_ = 1.04, *p* = 0.3214; *t*_11_ = 0.42, *p* = 0.6850).

### Experiment 3: Propranolol and cocaine—USV emission and CPP

A total of 39,527 50-kHz calls and 176 22-kHz calls were identified within the 192 recording sessions (i.e., 8 test days, 24 rats). Most of the 22-kHz calls (i.e., 162/176) were confined to two test sessions, respectively, propranolol–saline and propranolol–cocaine, and there was no apparent relationship with drug condition.

**Figure 4.**
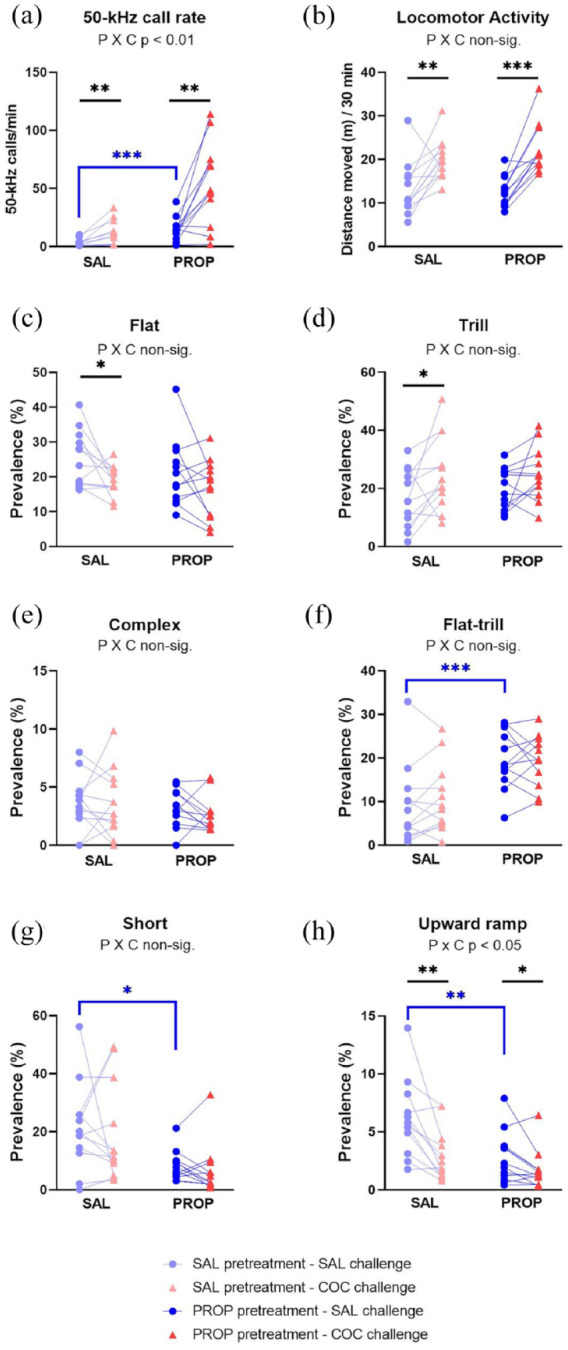
50-kHz call rate, locomotor activity, and selected subtypes during conditioning sessions for Experiment 3. *Y*-axes show the 50-kHz call rate (a), locomotor activity (b), and the percentage prevalence of flat (c), trill (d), and other selected call subtypes (e–h). The 24 rats were randomly assigned to two groups, respectively, receiving either saline or propranolol pretreatment on each CPP conditioning day (n = 12/group). Each rat received four pairs of daily conditioning sessions. On alternate days, they were injected pre-session with saline and cocaine 10 mg/kg IP. Data were collapsed across days within each drug condition. Each line represents an individual rat. “P × C” refers to a propranolol × cocaine interaction (ANOVA or nonparametric equivalent). *p < 0.05 (i.e., trend), **p < 0.01, ***p < 0.001 vs. corresponding saline condition (independent *t*-test, or Wilcoxon or Mann–Whitney U test).

Both propranolol and cocaine, when given alone, significantly increased the 50-kHz call rate (respectively MW *p* = 0.0005; W *p* = 0.0022; Figure 4(a)). Propranolol, rather than blunting this call rate-stimulating effect of cocaine, potentiated it (cocaine–saline difference scores, Welch’s *t*-test *p* = 0.0073). By contrast, although LMA was stimulated by cocaine (t_11_ = 3.30, *p* = 0.0070; Figure 4(b)), propranolol did not alter this effect (PROP × COCAINE F_1,22_ = 1.15, *p* = 0.2954), nor did it increase LMA when given alone (t_22_ = 0.27, *p* = 0.7876).

Analyses of the relative prevalence of individual call subtypes showed that neither drug significantly altered flat or trill call percentages. However, cocaine when given alone tended to suppress flat calls (t_11_ = 2.56, *p* = 0.0265; Figure 4(b)), and promote trills (t_11_ = 2.32, *p* = 0.0404; Figure 4(d)). In terms of non-flat/non-trill subtypes, cocaine given alone significantly decreased upward ramp calls (W *p* = 0.0047; Figure 4(h)), whereas propranolol given alone exerted multiple effects. Specifically, propranolol significantly decreased upward ramps (MW *p* = 0.0056; Figure 4(h)) and tended to decrease short calls (MW *p* = 0.0153; Figure 4(g)), whereas it significantly increased the flat–trill subtype (MW *p* = 0.0032; Figure 4(f)), trill with jumps (MW *p* = 0.0054; Supplemental Figure 1(C)) and composite calls (MW *p* = 0.0037; Supplemental Figure 1(C)). No significant interaction was detected between propranolol and cocaine for any 50-kHz call subtype, including flat and trill calls (ANOVA interactions, respectively: F_1,22_ = 0.31, *p* = 0.5830 and F_1,22_ = 0.68, *p* = 0.4182).

The locomotor stimulant effect of cocaine was not significantly correlated with any of the USV-related measures (i.e., 50-kHz call rate or the percent prevalence of any of the 50-kHz call subtypes).

### Experiment 4 Phase 1: Effects of acute cocaine in drug-naïve animals (days 1 and 2)

Phase 1 of this experiment determined the acute effects of cocaine (10 mg/kg) in drug-naïve animals (days 1 and 2) and after repeated cocaine exposure (days 9–11). On days 1 and 2, the group allocated to subsequently receive repeated cocaine was tested once with cocaine and once with saline (n = 10/group), whereas the control (repeated saline) group was tested twice with saline.

**Figure 5.**
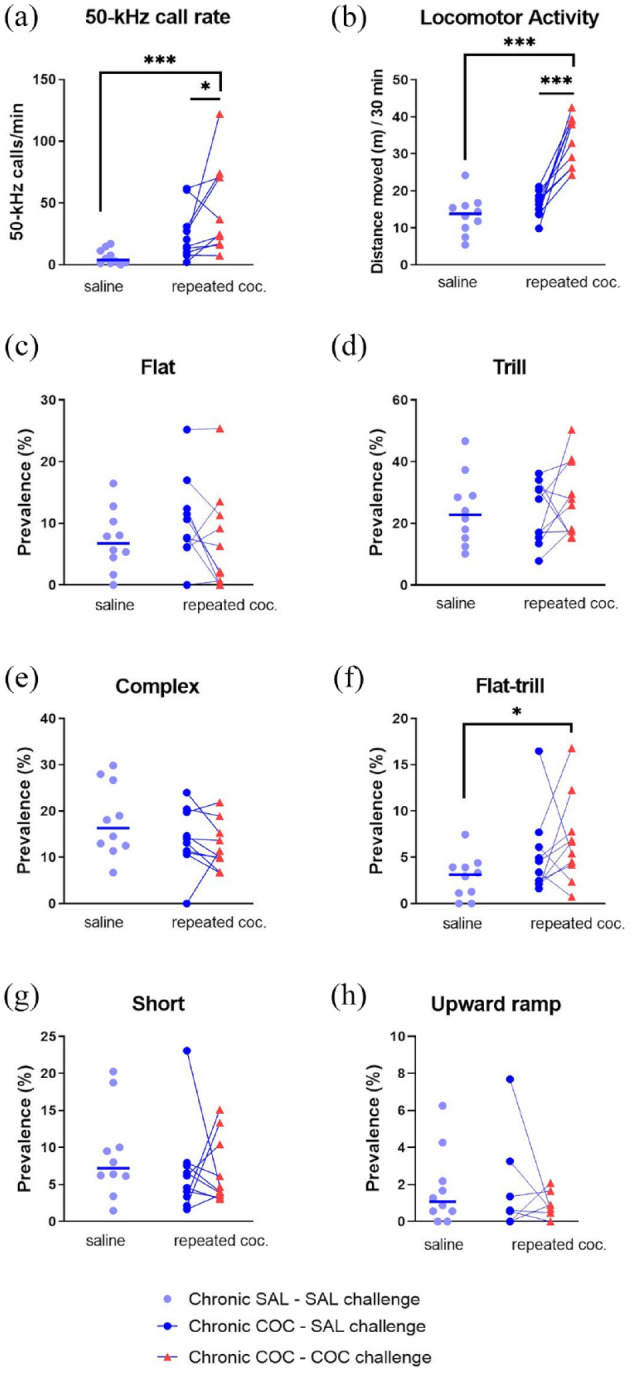
Call rate, locomotor activity, and selected call subtypes for the two initial test days of Experiment 4 (Experiment 4 Phase 1, days 1 and 2). *Y*-axes show the 50-kHz call rate (a), locomotor activity (b), and the percentage prevalence of flat (c), trill (d), and other selected call subtypes (e–h). The 20 rats were randomly assigned to two groups. The control group was tested with saline on both days 1 and 2, whereas the repeated cocaine group was tested once with saline and once with cocaine, in counterbalanced order. Horizontal lines indicate the median for panels a, and e–h, for the saline group, and the mean for panels b–d. *p < 0.05 (i.e., trend), **p < 0.01, ***p < 0.001 versus corresponding saline condition (paired or independent *t*-test or Mann–Whitney U test).

On these two initial days, a total of 4807 50-kHz calls and 16 22-kHz calls were sampled. Cocaine significantly increased the 50-kHz call rate relative to the saline control group (MW *p* = 0.0007; Figure 5(a)) while also increasing LMA (t_18_ = 7.45, *p* < 0.0001; Figure 5(b)). However, cocaine did not significantly alter the percentage of flat calls (COCAINE main effect F_1,17_ = 3.55, *p* = 0.0768) or trills (t_18_ = 0.72, *p* = 0.4829 and t_9_ = 1.01, *p* = 0.3376; Figure 5(c) and (d)), or the relative prevalence of any other 50-kHz call subtype (*p* > 0.1 for all subtypes; Figure 5(e)–(h), Supplemental Figure 1(D)). The only trend observed was an apparent cocaine-induced increase in flat–trill calls (MW *p* = 0.0233; Figure 5(f)).

### Experiment 4 Phase 1: Sensitization to acute cocaine effects (days 9–11)

**Figure 6.**
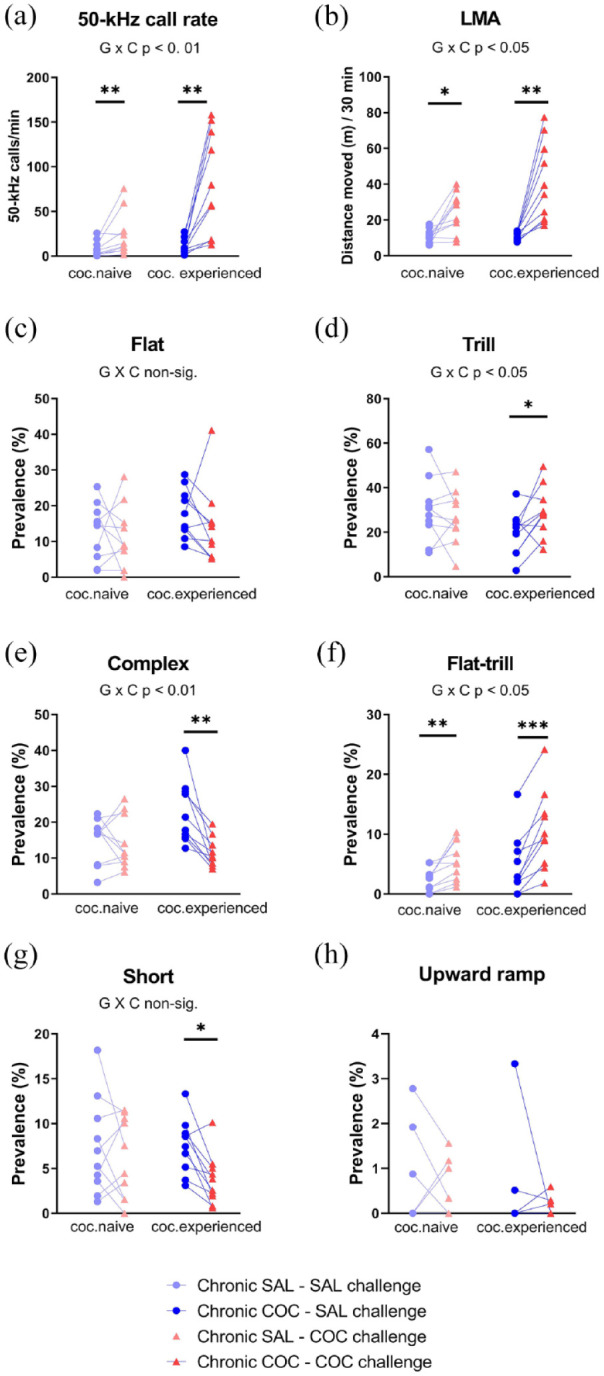
Call rate, locomotor activity (LMA), and selected call subtypes after repeated cocaine exposure (Experiment 4 Phase 1, days 9–11). Y-axes show the 50-kHz call rate (a), locomotor activity (b), and the percentage prevalence of flat (c), trill (d), and other selected call subtypes (e–h). Before days 9–11, subjects had been tested daily, either with saline (cocaine naïve, n = 10) or with saline and cocaine on alternate days (cocaine experienced, n = 10). On days 9–11, rats in both groups were each tested twice with saline (data pooled) and once with cocaine, in a counterbalanced order. In panel h, some rats had zero values after both saline and cocaine challenge. *p < 0.05 (i.e., trend), **p < 0.01, ***p < 0.001 versus acute saline condition (paired *t*-test or Wilcoxon test).

On days 9–11, each rat was tested twice with saline and once with cocaine. A total of 7163 50-kHz calls and zero 22-kHz calls were sampled in the 60 test sessions (i.e., 3 days, 20 rats). Cocaine increased the 50-kHz call rate in both groups, that is, in the control group that was receiving cocaine for the first time, as well as in the group that had already received several cocaine tests (W: *p* = 0.0093 and *p* = 0.0051, respectively; Figure 6(a)). This rate-increasing effect of cocaine was greater in the cocaine-experienced group (nonparametric GROUP × COCAINE interaction: MW *p* = 0.0065). Sensitization also appeared to occur to cocaine’s locomotor stimulant effect (GROUP × COCAINE F_1,18_ = 5.05, *p* = 0.0374; Figure 6(b)). Lastly, the two groups did not differ significantly in terms of 50-kHz call rates during saline sessions (MW *p* = 0.2561; Figure 6(a)); hence, cocaine-conditioned 50-kHz call emission was not detected.

Cocaine did not convincingly change the relative prevalence of flat or trill calls, as seen in Figure 6(c) and (d). Thus, there were no significant COCAINE main effects (respectively: F_1,17_ = 3.55, *p* = 0.0768 and F_1,18_ = 1.45, *p* = 0.2439) or GROUP × COCAINE interactions (F_1,17_ = 1.59, *p* = 0.2240 and F_1,18_ = 5.24, *p* = 0.0344). Acute cocaine did, however, alter the relative prevalence of some other 50-kHz call subtypes (Figure 6(e)–(g)). Specifically, with the two chronic groups pooled, cocaine significantly decreased the percentage of complex calls (W *p* = 0.0090) and increased the flat–trill subtype (W *p* = 0.0001); it also tended to decrease short calls (W *p* = 0.0228) and to increase unclear calls (W *p* = 0.0304). There were few if any differences between the cocaine-naïve (control) and cocaine-experienced rats (Figure 6(e)–(g)), with statistical trends only for complex and flat–trill calls (nonparametric GROUP × COCAINE *p* = 0.0126 and 0.0494, respectively).

### Experiment 4 Phase 2: Propranolol pretreatment with cocaine and amphetamine challenge—USV emission and LMA

Propranolol pretreatment with cocaine and amphetamine challenge—USV emission: The main goal here was to investigate potential interactions between propranolol and cocaine, with amphetamine intended to serve as a positive control (see Introduction).

Here, a total of 132 22-kHz and 24,377 50-kHz calls were sampled. The 22-kHz vocalizations were mostly emitted by two rats, unrelated to the drug condition. In the initial analysis, we detected no residual effect of drug history from Phase 1 (i.e., repeated saline vs. cocaine), on any behavioral measure. Accordingly, the two groups from Phase 1 were merged, giving n = 20. One high outlier was detected (Grubb’s test) and excluded from this measure.

**Figure 7.**
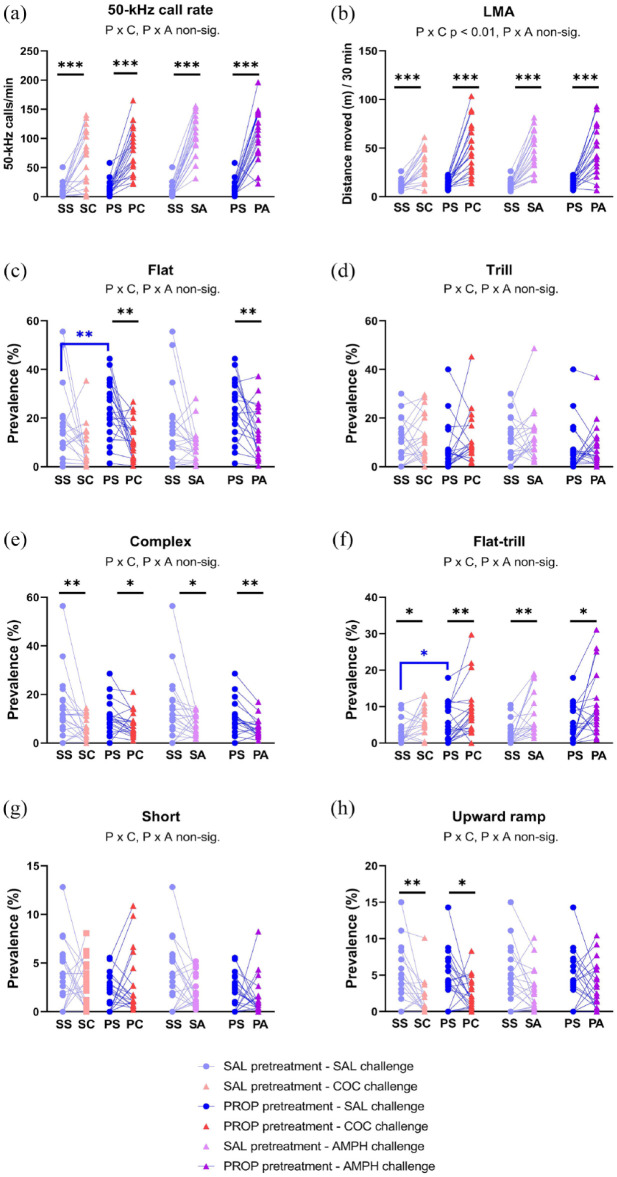
Interactions between propranolol and cocaine or amphetamine (Experiment 4 Phase 2). Y-axes show the 50-kHz call rate (a), locomotor activity (b), and the percentage prevalence of flat (c), trill (d), and other selected call subtypes (e–h). The x-axes show the acute cocaine challenge on the left (red triangles), and the amphetamine challenge on the right (purple triangles). The saline (control) pretreatment is shown as light blue circles, and propranolol pretreatment as dark blue circles. Rats from the two Phase I groups (i.e., repeated saline or cocaine) were pooled, hence n = 20. Each rat received each pretreatment–treatment combination, in a counterbalanced order. *p < 0.05 (trend), **p < 0.01, ***p < 0.001, versus corresponding vehicle/saline conditions (paired or independent *t*-tests, or nonparametric equivalent). In each panel, interactions between propranolol and cocaine or amphetamine are indicated by “P × C” and “P × A,” respectively.

Cocaine increased the 50-kHz call rate overall (W *p* = 0.0003; Figure 7(a)), as in Phase 1. Although the cocaine effect did not differ significantly between the two chronic groups (nonparametric PROP × COCAINE *p* = 0.5453), it was significant only in the chronic cocaine group (W *p* = 0.0093 vs . *p* = 0.0166 trends in the chronic saline group). Amphetamine, when given alone, also increased the 50-kHz call rate (W . *p* < 0.0001; Figure 7(a)). As seen in Figure 7(a), propranolol did not impact the call rate-increasing effects of either cocaine (nonparametric PROP × COCAINE *p* = 0.4221) or amphetamine (nonparametric PROP × AMPH *p* = 0.7510). Lastly, propranolol did not affect the call rate alone (W *p* = 0.0674).

Flat calls were suppressed by both cocaine and amphetamine (Figure 7(c)). Thus, cocaine significantly decreased the percentage of flat calls (COCAINE main effect F_1,18_ = 11.09, *p* = 0.0037; high outlier excluded), with no significant PROP × COCAINE interaction (F_1,18_ = 1.77, *p* = 0.2065). Amphetamine produced comparable results (AMPH main effect F_1,18_ = 8.91, *p* = 0.00079; high outlier excluded; PROP × AMPH F_1,18_ = 0.39, *p* = 0.5422). When given alone, neither cocaine nor amphetamine detectably altered flat calls (t_19_ = 1.87, *p* = 0.0766; t_19_ = 1.96, *p* = 0.0646, respectively). Propranolol, when given alone, significantly increased the percentage of flat calls (t_19_ = 2.93, *p* = 0.0086; Figure 7(c)).

The relative prevalence of trills calls appeared unaffected by cocaine and amphetamine, as seen in Figure 7(d) (COCAINE F_1,18_ = 2.87, *p* = 0.1075; PROP × COCAINE F_1,18_ = 2.87, *p* = 0.1339; AMPH F_1,18_ = 0.38, *p* = 0.5440; PROP × AMPH F_1,18_ = 0.0015, *p* = 0.9693; high outlier excluded). None of the three drugs, given alone, altered this measure (propranolol, cocaine, and amphetamine respectively: t_19_ = 1.78, *p* = 0.0905; t_19_ = 0.77, *p* = 0.4487; t_19_ = 0.92, *p* = 0.3643).

Cocaine changed the relative prevalence of certain non-flat/non-trill call subtypes, and amphetamine tended to mimic these effects (Figure 7(e)–(h), Supplemental Figure 1(F)). These effects, which were largely as seen in Phase 1 (days 9–11), were not altered by propranolol (all PROP × COCAINE and PROP × AMPH interactions were non-significant). Specifically, cocaine alone significantly decreased complex calls (W *p* = 0.0079; Figure 7(e)) and upward ramp calls (W *p* = 0.0052; Figure 7(h)), and tended to increase flat–trill calls (Figure 7(f)), trill with jumps and composite calls (respectively: W *p* = 0.0262, 0.0311 and 0.0148; Supplemental Figure 1(F)). Similarly, amphetamine alone significantly increased the latter three subtypes (respectively: W *p* = 0.00102, 0.0036, and 0.0064; Figure 7(f), Supplemental Figure 1(F)).

Propranolol when given alone also affected some of these non-flat/non-trill calls: it tended to increase the percentage of flat–trill (W *p* = 0.0113; Figure 7(f)) and composite calls (medians 0.4% vs. 3.3%, W *p* = 0.0229) and significantly decreased the “unclear” call subtype (medians 19% vs. 12%, W *p* = 0.0040). No significant interactions occurred between propranolol and either cocaine or amphetamine, although propranolol tended to inhibit amphetamine’s ability to increase the trill with jumps subtype (nonparametric PROP × AMPH interaction *p* = 0.0479).

LMA was markedly increased by cocaine and amphetamine when either drug was given alone (respectively: t_19_ = 7.07, *p* < 0.0001 and t_19_ = 6.96, *p* < 0.0001), or in combination with propranolol (Figure 7(b)). Propranolol given alone produced a much smaller (27%) but significant increase in LMA (t_19_ = 3.15, *p* = 0.0053). Propranolol also significantly increased the locomotor stimulant effect of cocaine (PROP × COCAINE F_1,19_ = 9.04, *p* = 0.0072), whereas no such synergy was seen with amphetamine (PROP × AMPH F_1,19_ = 0.03, *p* = 0.8586; Figure 7(b)).

### Experiment 4 Phase 3: Effects of propranolol and cocaine during conditioning sessions

With all 20 rats having now received multiple cocaine exposures, the aim was to test for the effects of propranolol on cocaine-induced USV emission and LMA, as well as on the acquisition of cocaine CPP. In total, 21,615 50-kHz calls and 71 22-kHz calls were sampled. The 22-kHz calls were emitted by 13/20 rats in 13 of the 160 conditioning sessions; most of these calls (59/71) were made on conditioning days 4 and 5. There was no clear relationship between 22-kHz call emission and drug condition.

**Figure 8.**
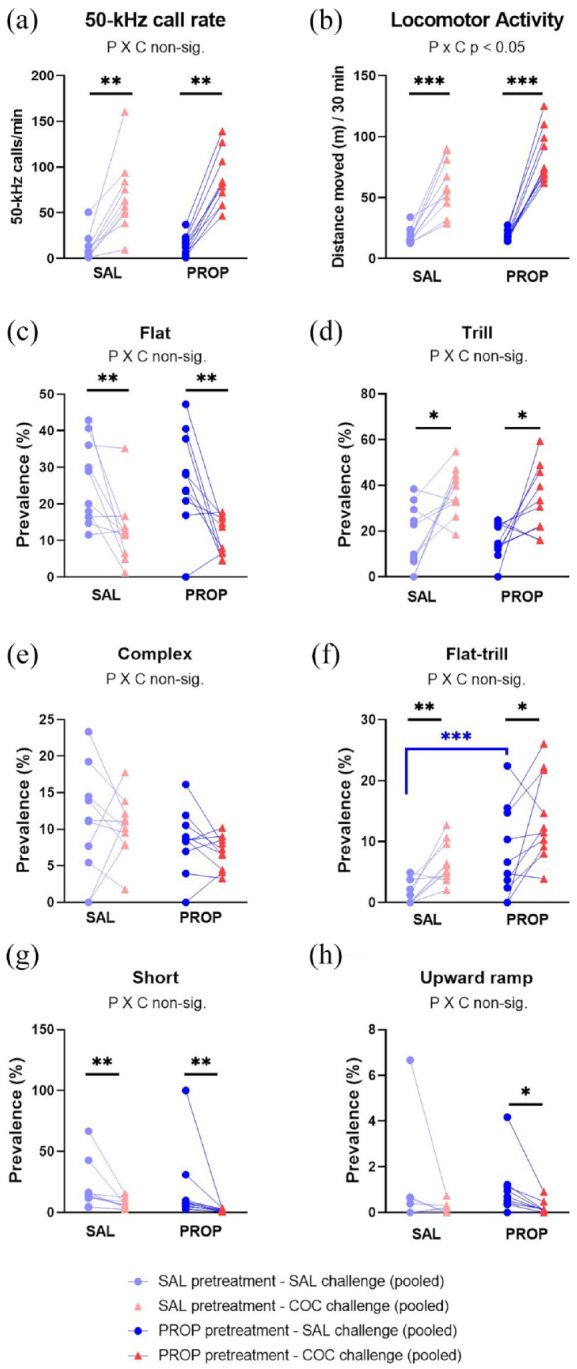
50-kHz call rate, LMA, and selected subtypes for Experiment 4 Phase 3 conditioning days. Y-axes show the 50-kHz call rate (a), locomotor activity (b), and the percentage prevalence of flat (c), trill (d), and other selected call subtypes (e–h). After Phase 2, the 20 rats were randomly assigned to two groups, that is, receiving saline versus propranolol pretreatment on the 8 CPP conditioning days (SAL and PROP, respectively, n = 10 rats). Before a daily session, each rat received an injection of saline or cocaine 10 mg/kg IP, alternating across days. Data were collapsed across the four saline sessions and separately across the four cocaine sessions. *p < 0.05 (trend), **p < 0.01, ***p < 0.001, versus corresponding saline condition (paired or independent *t*-tests, or nonparametric equivalent). In each panel, an interaction between propranolol and cocaine is indicated by “P × C.”

Cocaine again markedly increased the 50-kHz call rate, both with and without propranolol pretreatment (W *p* = 0.0051 for both; Figure 8(a)). Propranolol did not detectably alter this stimulant effect (nonparametric PROP × COCAINE interaction: *p* = 0.1306) and did not change 50-kHz call rate when it was administered alone (MW *p* = 0.4057; Figure 8(a)). Once again, cocaine also stimulated LMA (t_9_ = 6.24, *p* = 0.0002; Figure 8(b)), an effect possibly accentuated by propranolol (PROP × COCAINE F_1,18_ = 6.70, *p* = 0.0185). The locomotor stimulant effect of cocaine was not significantly correlated with any of the USV-related measures (i.e., 50-kHz call rate or the percent prevalence of any of the 50-kHz call subtypes).

In this final phase, cocaine for the first time both suppressed flat calls and promoted trill calls (COCAINE main effects, respectively: F_1,17_ = 22.59, *p* = 0.0002, with high outlier omitted; F_1,18_ = 16.71, *p* = 0.0007; Figure 8(c) and (d)), and these two effects on relative prevalence were unaltered by propranolol pretreatment (PROP × COCAINE F_1,17_ = 0.03, *p* = 0.8665 and F_1,18_ = 0.05, *p* = 0.8209, respectively). The relative prevalence of flat and trill call were unaffected by propranolol given alone (t_18_ = 0.14, *p* = 0.8902 and t_18_ = 0.35, *p* = 0.7337; Figure 8(c) and (d)).

Non-flat/non-trill call subtype emission was altered by cocaine and possibly by propranolol, as shown in Supplemental Figure 1(G) and Figure 8(e)–(h). Cocaine given alone significantly decreased the percentage of short calls (W *p* = 0.0051; Figure 8(g)) and increased the percentage of flat–trill calls (W *p* = 0.0093; Figure 8(f)). Propranolol given alone exerted no significant effects, but tended to increase flat–trill calls (W *p* = 0.0306, Figure 8(f)), and also split and composite calls (respectively: W *p* = 0.0413 and *p* = 0.0155; Supplemental Figure 1(G)), and tended to decrease “unclear” calls (W *p* = 0.0281; Supplemental Figure 1(G)). The only significant PROP × COCAINE interaction occurred for the trill with jumps call subtype (*p* = 0.0081); cocaine significantly increased the relative prevalence of this call subtype, but only in the presence of propranolol (*p* = 0.0051; Supplemental Figure 1(G)).

As in Experiment 3, the locomotor stimulant effect of cocaine was not significantly correlated with any of the USV-related measures (i.e., 50-kHz call rate or the percent prevalence of any of the 50-kHz call subtypes).

### Experiments 3 and 4.3: Propranolol pretreatment and cocaine CPP

**Figure 9.**
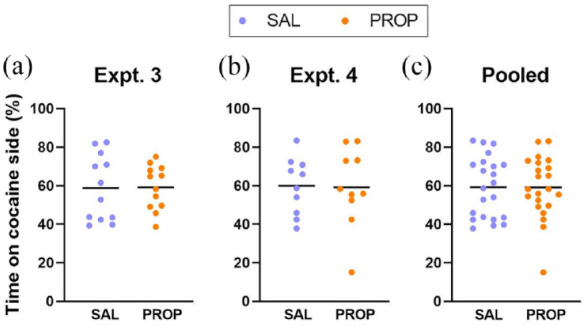
CPP tests in Experiment 3 and Phase 3 of Experiment 4. The y-axes show the percentage of time spent on the cocaine-associated side. Each circle shows an individual rat (n = 12 and 10/group for panels a and b, respectively). Panel c shows the pooled data from the two experiments, with n = 22/group. Horizontal lines indicate the mean time spent on the cocaine side for panels a–c.

In both experiments where cocaine CPP was tested, the rats only marginally preferred the drug-paired floor texture (Figure 9). In Experiment 3, cocaine produced a significant CPP when the saline and propranolol pretreatment groups were pooled (one-sample *t*-test vs. 50%: t_23_ = 3.09, *p* = 0.0052), and propranolol did not significantly alter cocaine CPP (t_22_ = 0.07, *p* = 0. 0.9457). However, cocaine failed to produce a significant CPP in either group taken alone (saline and propranolol, respectively: t_11_ = 1.78, *p* = 0.1023 and t_11_ = 2.76, *p* = 0.0185; Figure 9(a)). In Experiment 4, the apparent cocaine CPP represented only a statistical trend, even with the saline and PROP pretreatment groups pooled (t_19_ = 2.46, *p* = 0.0237; Figure 9(b)). However, one rat from the propranolol group exhibited a marked place aversion, spending only 15% of its time on the cocaine-paired side (Figure 9(b)). With this animal excluded, the overall cocaine CPP was significant (t_18_ = 3.63, *p* = 0.0019), and propranolol did not alter the CPP magnitude (t_17_ = 0.62, *p* = 0.5438).

To gain greater statistical power, the two CPP experiments (Experiments 3 and 4) were combined, after verifying that there were no significant inter-experiment differences (two-way ANOVA: *p* > 0.5 for all). With this expanded data set (i.e., 43 subjects, excluding the rat showing extreme place aversion), cocaine produced a significant CPP (t_42_ = 4.76, *p* < 0.0001; Figure 9(c)), which was not significantly altered by propranolol (t_41_ = 0.45, *p* = 0.6530; Figure 9(c)). The cocaine CPP was clearer in the propranolol group (saline and propranolol pretreatment groups, respectively: t_21_ = 2.79, *p* = 0.0111 and t_20_ = 4.07, *p* = 0.0006).

The cocaine CPP magnitude in either of the two experiments was not significantly correlated with any of the USV-related measures (i.e., 50-kHz call rate or the percent prevalence of any of the 50-kHz call subtypes). There was no correlation between CPP and USV-related measures even when the two experiments were pooled.

## Discussion

### Main novel findings

Acute effects of cocaine on 50-kHz call emission have been widely reported, but among approximately 30 primary data papers, only five of these to our knowledge have reported the effects of IP cocaine administration on distinct 50-kHz call categories (Cordeiro et al., 2024; Kolosowska et al., 2023; Meyer et al., 2012; Sanchez et al., 2022; Tripi et al., 2017). The present analysis of cocaine-induced USV emission uses a much finer-grained (i.e., 14-call subtype) approach but also considerably extends the dosing regimen used in previous reports.

The main novel findings are as follows. In drug-naïve animals, IP cocaine administration did not detectably alter the 50-kHz call profile although it did, as expected, increase the 50-kHz call rate and LMA. After repeated exposure to the drug, acute cocaine injection increased the call rate more strongly, in line with previous reports, but also increased the relative prevalence of trill calls while suppressing flat calls, and even exerted effects on certain other 50-kHz call subtypes. When given alone, propranolol altered USV emission and increased the relative prevalence of two non-flat/non-trill call subtypes. While propranolol tended to increase cocaine’s locomotor stimulant effect, as previously reported by others, this drug did not detectably attenuate cocaine CPP or the effects of cocaine on USV emission.

### Cocaine experience resulted in an enhanced 50-kHz call rate-stimulating effect, with no detectable conditioned response

In rats that were initially drug-naive, IP cocaine injection tended to increase the 50-kHz call rate, consistent with previous studies (Cordeiro et al., 2024; Kolosowska et al., 2023; Meyer et al., 2012; Sanchez et al., 2022; Simmons et al., 2018; Tripi et al., 2017). However, with repeated treatment, both tolerance and sensitization have been reported (Ahrens et al., 2009; Maier et al., 2010, 2012; Mu et al., 2009; Sohn et al., 2022; Williams and Undieh 2010, 2016). These different outcomes presumably reflect procedural differences, for example, passive- versus self-administration, IP versus IV route of administration, cocaine dose, rat stock, etc.

In the present study, we found sensitization to cocaine’s stimulant effects on 50-kHz call rate and LMA, in the absence of conditioned drug effects. These findings are consistent with previous USV studies featuring a similar regimen of repeated cocaine administration (Mu et al., 2009; Tripi et al., 2017), although in the latter study, the apparent sensitization was confounded by time. By way of comparison, we have previously observed that morphine’s ability to stimulate 50-kHz calling emerged only after repeated intermittent morphine treatment, and also occurred without any detectable conditioned stimulant effect (Best et al., 2017).

### Cocaine, given alone, promoted trills and suppressed flat calls, but only after repeated experience

In drug-naïve animals, neither cocaine nor amphetamine clearly promoted trills or suppressed flat calls (Experiments 1, 3, and 4 Phase 1). The lack of initial drug effects might seem surprising, as we previously reported that in rats with little previous drug experience, *intravenous* cocaine and IP amphetamine administration both promoted trills at the expense of flat calls (Wright et al., 2010, 2012b, 2013). Several explanations for our negative findings are possible. First, in our three previous studies, rats were pre-screened three times with amphetamine, potentially producing some sensitization. This prescreening was done to identify and reject low-callers (20%–40% of the animals). By contrast, low-calling rats in the present study were not pre-screened or excluded. However, when we re-analyzed our data after the *post hoc* exclusion of amphetamine session low-callers, no further effects of either amphetamine or cocaine were revealed. Second, cocaine possibly exerts USV-related effects through different mechanisms, depending on whether it is injected IP or IV—as seen in the CPP paradigm (see Introduction). Third, cocaine can exert both rewarding and aversive effects, when it is experimenter-delivered and even self-administered (Colechio and Grigson, 2014; Ettenberg et al., 2015).

The absence of a clear cocaine effect on trill versus flat call prevalence in cocaine-*inexperienced* rats is broadly consistent with the literature, although procedural differences make comparison difficult. In particular, of the four published analyses of 50-kHz call subtype emission following IP cocaine administration, all employed fewer call categories and in particular tended to use broader definitions of “trill” calls (see below). In the first of these, Meyer et al. (2012) distinguished only two call categories (FM and flat), and cocaine (10 mg/kg IP) increased the emission of both call types; however, *relative* prevalence data were not presented. Sanchez et al. (2022) reported that cocaine (20 mg/kg IP) increased the emission of their four call categories non-selectively, whereas the same group (Cordeiro et al., 2024) subsequently reported a preferential increase in trill and step calls; importantly, however, saline tests always preceded cocaine tests in the latter study. Lastly, Tripi et al. (2017) used a more extensive seven-way categorization scheme but reported only cocaine-session data.

With extended drug testing, cocaine did eventually promote trills over flat calls (Experiment 4 Phase 3). This occurred after an intermediate stage during which the drug suppressed flat calls without enhancing trill calls (Experiment 4 Phase 2). These call subtype-specific effects of cocaine emerged more slowly, and were less striking, than the drug’s marked enhancement of the 50-kHz call rate. Amphetamine, tested in Phase 2 of the same experiment, did not increase trill call prevalence but, like cocaine, significantly suppressed flat calls. It is possible that prior cocaine exposure facilitated the latter effect, as it was not seen in cocaine-inexperienced rats (Experiment 1).

### Cocaine, given alone, altered the emission of lesser-studied 50-kHz subtypes

In our published and unpublished work, test drugs typically altered the relative prevalence of only flat and trill calls. By contrast, in the present study, several other call subtypes were altered by cocaine and/or amphetamine (see Table 2). Cocaine, when given alone at 10 mg/kg, significantly decreased the relative prevalence of complex, upward ramp, and short calls while promoting flat–trill calls. However, these effects were not detected in all experiments, presumably reflecting differences in drug history, insufficient statistical power, or other factors. In drug-experienced rats, cocaine and amphetamine shared two effects on 50-kHz call subtypes: an increase in flat–trill call prevalence, and a suppression of flat calls (Experiment 4 Phase 2).

Where the same fine-grained call categorization scheme has been applied in past drug studies, few effects have been detected on non-flat/non-trill call subtypes. However, as in the present study, amphetamine did promote trill with jumps in two studies (Wright et al., 2012b, 2012a, Supplemental Table S4; but see Wright et al., 2010). As with cocaine (present study), morphine has also been shown to acutely suppress flat and short calls *only* in animals with extensive drug experience (Best et al., 2017).

### Cocaine CPP

In both Experiments 3 and 4, cocaine produced only a weak CPP. This was especially unexpected in Experiment 4, given the rats’ extensive cocaine history (Harris et al. 2001; Le Pen et al., 1998; Lett, 1989; Shippenberg and Heidbreder, 1995a, 1995b). While the present dose (10 mg/kg IP) has produced a clear CPP in most published studies (reviewed by Bardo et al., 1995), CPP at this dose was weak or even absent in others (Allen et al., 2007; Nomikos and Spyraki, 1998; Pelloux et al., 2009; Yates et al., 2021). Furthermore, substantial inter-individual differences have been noted, with some individual rats either not expressing a CPP (Niedzielska-Andres et al., 2019; Seymour and Wagner, 2008) or even avoiding the cocaine-paired context (Atehortua Martinez et al., 2022; Yates et al., 2021)—as in the present study.

### Propranolol did not blunt cocaine effects on the 50-kHz call profile or cocaine CPP

As explained earlier (Introduction), we anticipated that cocaine would promote trill calls at the expense of flat calls, and that propranolol would counteract these effects as well as inhibit the acquisition of cocaine CPP. However, propranolol failed to block any of these effects even though it was clearly behaviorally active (see below). Cocaine’s effects on flat versus trill call prevalence were most evident in rats with an extended cocaine history (Experiment 3 Phase 3), and here there was no sign of a propranolol × cocaine interaction.

The literature on propranolol and cocaine CPP is, to our knowledge, limited to two studies (Bernardi et al., 2006; Otis and Mueller, 2011); in both, propranolol was administered only on the test day and was found to prevent CPP expression. In the present study, we asked instead whether propranolol would prevent the *acquisition* of cocaine CPP. Although cocaine produced only a weak CPP (see above), when the relevant data were pooled (i.e., from Experiments 3 and 4), a highly significant CPP was seen even in the animals pretreated with propranolol, and no propranolol × cocaine interaction was detected. Hence, the rewarding effect of cocaine in this paradigm does not appear to depend on beta-adrenergic receptors.

Propranolol also failed to blunt any other behavioral effects of cocaine. Exceptionally and for unclear reasons, in Experiment 3 propranolol significantly *augmented* cocaine-induced calling. In the same experiment, propranolol did not potentiate the locomotor stimulant effect of cocaine, although locomotor synergy was subsequently observed in Experiment 4, in line with a previous report (Harris, 1996).

### Propranolol given alone was behaviorally active on some measures

The dose of propranolol used in the present study (10 mg/kg IP) was chosen because it profoundly altered the 50-kHz call profile in rats challenged with amphetamine (Wright et al., 2012b). This dose, and even lower doses, has also been shown to be active in several other behavioral assays (e.g., Harris, 1996; O’Donnell, 1997; Kleven and Koek, 1997). In a previous study investigating the effects of propranolol on adult rat USV emission, no effects were detected (Hoffmeister et al., 2022). However, in this earlier study, the dose was lower (3 mg/kg IP), only two 50-kHz call categories were used, and the rats were genetically modified (i.e., Pink1 knockout). In the present study, since propranolol did not attenuate the behavioral effects of cocaine (Experiments 3 and 4), it is important to note that it was behaviorally active in the same experiments, mainly by increasing the relative prevalence of flat–trill and composite call subtypes.

Propranolol given alone produced other statistically significant, but sporadic, behavioral effects (see Table 2). Given that propranolol is a receptor antagonist, or even an inverse agonist (Chidiac et al., 1994), a possible reason for these mixed effects is that the degree of noradrenergic tone differed between experiments.

### Propranolol and amphetamine’s abilities to promote trills and suppress flat calls

In our previous study (Wright et al., 2012b), amphetamine promoted trill calls at the expense of flat calls, and propranolol appeared to counteract this effect, to the extent that trill calls were virtually abolished in the propranolol/amphetamine condition. However, in this earlier study, the effects of propranolol alone could not be determined since call rates were too low. In the present study, amphetamine did not produce the anticipated promotion of trill versus flat calls, and hence we were unable to *directly* test for a propranolol × amphetamine interaction (Experiment 4 Phase 2). The present findings do, however, indirectly suggest that propranolol can prevent amphetamine from promoting trills and suppressing flat calls, given that propranolol alone did not consistently affect trill call prevalence, and had little if any effect on flat call prevalence. Our findings therefore support a true propranolol × amphetamine interaction. By contrast, *cocaine* acted independently of beta-adrenergic receptor stimulation to promote trills at the expense of flat calls (Experiment 4 Phase 3).

### Independence of USV-related versus other behavioral measures

Rat USVs are attracting attention in part because 50-kHz call-related measures appear unique, in that they are only loosely related to other behavioral outputs such as LMA (Burke et al., 2017; Cordeiro et al., 2024), CPP magnitude (Ahrens et al., 2013; Knutson et al., 1999; Meyer et al., 2012; Taracha et al., 2014; Wright et al., 2012a, 2013), and discriminative stimulus drug effects (Wright et al., 2013). In the present study, LMA and 50-kHz call rate were both stimulated by cocaine, but these effects were dissociated by propranolol across experiments. We also observed that in drug-naïve animals, cocaine increased the 50-kHz call rate without altering the 50-kHz call profile, thus adding to existing evidence that these measures are independent (e.g., Sundarakrishnan and Clarke, 2022).

### Possible relationship of 50-kHz call subtypes to positive affect

In adult rats, drugs and other stimuli that would be expected to produce a positive emotional state increase the relative prevalence of trill versus flat calls, and on this basis, we have proposed that trill calls, or the trill–flat relationship, may serve as a marker for positive affect (see Introduction). In the present study, cocaine did not promote trills or suppress flat calls in animals having little or no prior drug experience. However, it appears unknown whether cocaine would produce euphoria in drug-naïve, psychiatrically normal, *human* subjects; all reports of cocaine-induced euphoria in human subjects, to our knowledge, concern either depressed individuals or individuals with significant cocaine experience, gained either through abuse or recreational use. In the present study, cocaine acutely shifted the trill/flat balance in animals that had repeatedly experienced the drug. Although cocaine’s subjective effects appear uninvestigated after IP injection in human subjects, the drug is euphorigenic even when taken through intranasal and oral routes (Johanson and Fischman, 1989; Smith and Griffiths, 2001) which provide slow absorption rates similar to IP injection in animals (Allain et al., 2015).

### Strengths, limitations, and other methodological considerations

Strengths of the present study include the large number of calls analyzed, the chronic drug component, the extensive replications within and across experiments, and the fine-grained approach to 50-kHz call subtype analysis. This detailed subtype analysis was useful in two main ways. First, this approach revealed that upon chronic intermittent exposure, cocaine can promote trill calls (i.e., as narrowly defined by Wright et al., 2010), whereas such an effect would probably have been missed, if multiple frequency-modulated call categories had been pooled. Second, a number of non-flat/non-trill call subtypes were affected by cocaine, amphetamine, or propranolol; these findings support previous evidence that individual 50-kHz call subtypes are not in general inter-correlated (Sundarakrishnan and Clarke, 2022) and that certain of these less prevalent call subtypes can be preferentially promoted or suppressed by drug challenge (discussed above). The potential biological significance of non-flat/non-trill subtypes calls for further investigation.

Limitations include the exclusive use of passive drug administration and male rats; while the latter facilitated comparison with our previous studies, it must be acknowledged that far less is known about USV emission in female rats and there are sex differences in terms of cocaine euphoria in humans (Evans and Foltin, 2010). Recent evidence suggests that cocaine’s effects on 50-kHz call subtype emission are not appreciably sex-dependent (Cordeiro et al., 2024), but the same may not be true for propranolol. A second limitation is that most of our behavioral tests used a single, albeit commonly used, dose of cocaine and propranolol. A third limitation is that drugs were given in the testing apparatus, whereas the effects of cocaine can depend on the setting, that is, home cage versus testing apparatus (Ahmed et al., 2020).

## Conclusions

The acute effects of cocaine on USV emission evolved over the course of repeated intermittent exposure. With sufficient exposure, cocaine promoted trill calls at the expense of flat calls—a shift previously observed with amphetamine and IV cocaine administration, after minimal exposure. To the extent that this call profile shift reflects positive affect, then repeated treatment would appear necessary for cocaine euphoria to emerge, at least after IP administration. Neither the cocaine-induced call profile shift nor cocaine place preference appeared dependent on beta-adrenergic receptors. The additional finding that propranolol given alone affected neither trill nor flat calls, however, strengthens our previous suggestion that amphetamine *does* depend on beta-adrenergic signaling for its trill-promoting effect (Wright et al., 2012b). More generally, the present findings support the utility of a fine-grained analysis of 50-kHz call subtypes.

## Supplemental Material

sj-docx-1-jop-10.1177_02698811241268894 – Supplemental material for Adult rat ultrasonic vocalizations and reward: Effects of propranolol and repeated cocaine administration Supplemental material, sj-docx-1-jop-10.1177_02698811241268894 for Adult rat ultrasonic vocalizations and reward: Effects of propranolol and repeated cocaine administration by Leyla Erden, Adithi Sundarakrishnan and Paul BS Clarke in Journal of Psychopharmacology

sj-docx-2-jop-10.1177_02698811241268894 – Supplemental material for Adult rat ultrasonic vocalizations and reward: Effects of propranolol and repeated cocaine administration Supplemental material, sj-docx-2-jop-10.1177_02698811241268894 for Adult rat ultrasonic vocalizations and reward: Effects of propranolol and repeated cocaine administration by Leyla Erden, Adithi Sundarakrishnan and Paul BS Clarke in Journal of Psychopharmacology

sj-docx-3-jop-10.1177_02698811241268894 – Supplemental material for Adult rat ultrasonic vocalizations and reward: Effects of propranolol and repeated cocaine administration Supplemental material, sj-docx-3-jop-10.1177_02698811241268894 for Adult rat ultrasonic vocalizations and reward: Effects of propranolol and repeated cocaine administration by Leyla Erden, Adithi Sundarakrishnan and Paul BS Clarke in Journal of Psychopharmacology

sj-docx-4-jop-10.1177_02698811241268894 – Supplemental material for Adult rat ultrasonic vocalizations and reward: Effects of propranolol and repeated cocaine administration Supplemental material, sj-docx-4-jop-10.1177_02698811241268894 for Adult rat ultrasonic vocalizations and reward: Effects of propranolol and repeated cocaine administration by Leyla Erden, Adithi Sundarakrishnan and Paul BS Clarke in Journal of Psychopharmacology
