# Supplementary material for: Adult rat ultrasonic vocalizations and reward: Effects of propranolol and repeated cocaine administration
Source: J Psychopharmacol. 2024 Aug 12;38(11):1025–41. doi: 10.1177/02698811241268894 (PMC11528876; doi:10.1177/02698811241268894)
Supplement: sj-docx-1-jop-10.1177_02698811241268894 – Supplemental material for Adult rat ultrasonic vocalizations and reward: Effects of propranolol and repeated cocaine administration [file sj-docx-1-jop-10.1177_02698811241268894.docx]

**Supplemental Table 1. Absolute rates for each 50-kHz call subtype for all experiments and drug conditions** Absolute rates are reported as calls/min. Abbreviations are: SAL, saline (i.e. a single session, or average of multiple sessions); COC, cocaine; PROP, propranolol; and AMPH, amphetamine. Where not stated, doses are cocaine 10 mg/kg, propranolol 10 mg/kg, and amphetamine 1 mg/kg. Call subtypes: CX complex, UR upward ramp, DR downward ramp, FL flat, SH short, SP split, SU step-up, SD step-down, MS multi-step, TR trill, FT flat-trill, TJ trill with jumps, IU inverted-U, CS composite, UC unclear, MI miscellaneous. The "n" refers to the number of rats per condition.
