# Supplementary material for: Adult rat ultrasonic vocalizations and reward: Effects of propranolol and repeated cocaine administration
Source: J Psychopharmacol. 2024 Aug 12;38(11):1025–41. doi: 10.1177/02698811241268894 (PMC11528876; doi:10.1177/02698811241268894)
Supplement: sj-docx-2-jop-10.1177_02698811241268894 – Supplemental material for Adult rat ultrasonic vocalizations and reward: Effects of propranolol and repeated cocaine administration [file sj-docx-2-jop-10.1177_02698811241268894.docx]

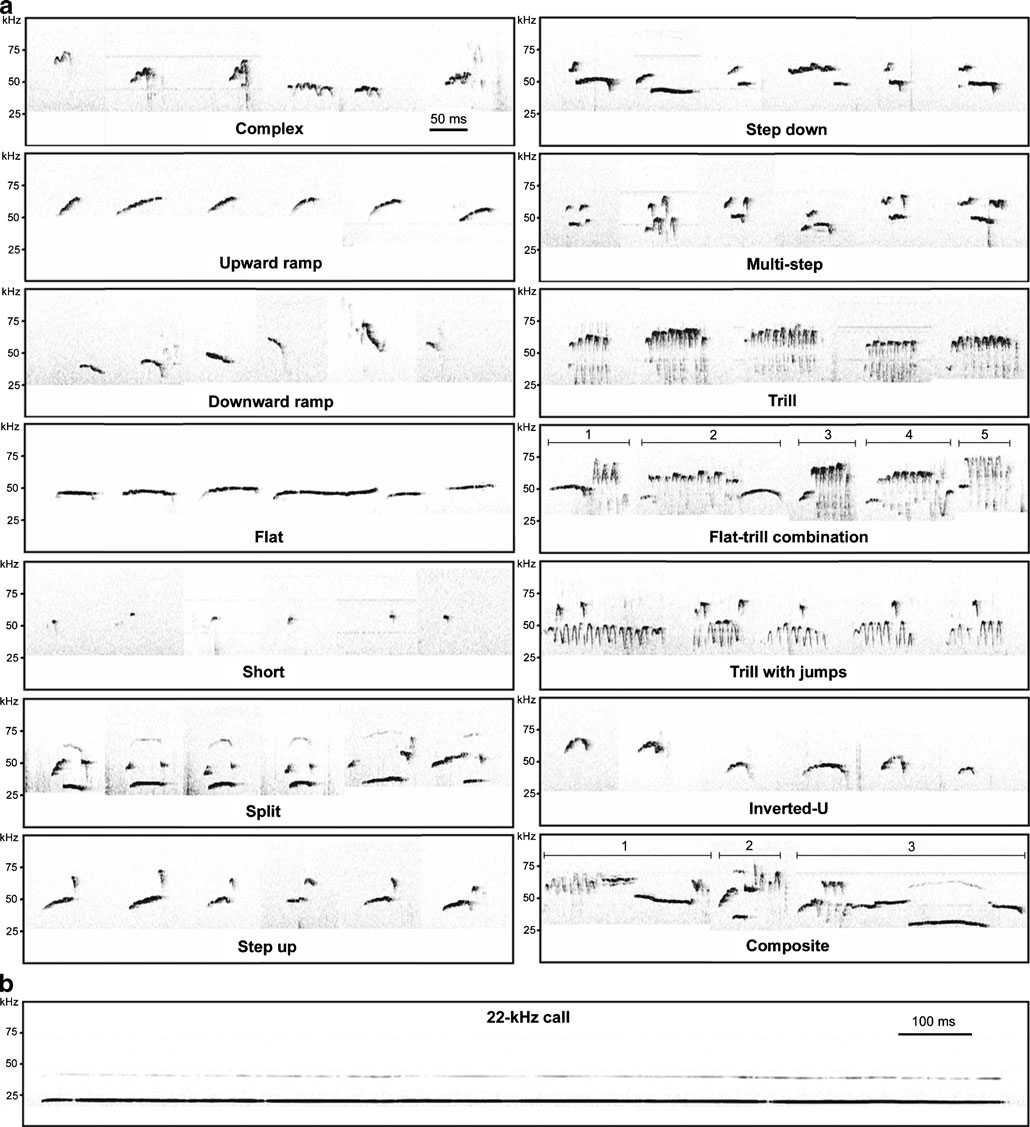


**Supplemental Figure 3.** **Spectrogram of USVs** Graphic representations of the calls as seen through processing software, of **(a)** all 14 50-kHz USV subtypes and **(b)** 22-kHz USVs. The y-axes show the frequency (kHz), and the x-axes represent time. The time scale for all *50-kHz calls* is indicated in the top left panel. Reproduced with permission from Wright et al (2010).
