# Supplementary material for: Adult rat ultrasonic vocalizations and reward: Effects of propranolol and repeated cocaine administration
Source: J Psychopharmacol. 2024 Aug 12;38(11):1025–41. doi: 10.1177/02698811241268894 (PMC11528876; doi:10.1177/02698811241268894)
Supplement: sj-docx-3-jop-10.1177_02698811241268894 – Supplemental material for Adult rat ultrasonic vocalizations and reward: Effects of propranolol and repeated cocaine administration [file sj-docx-3-jop-10.1177_02698811241268894.docx]

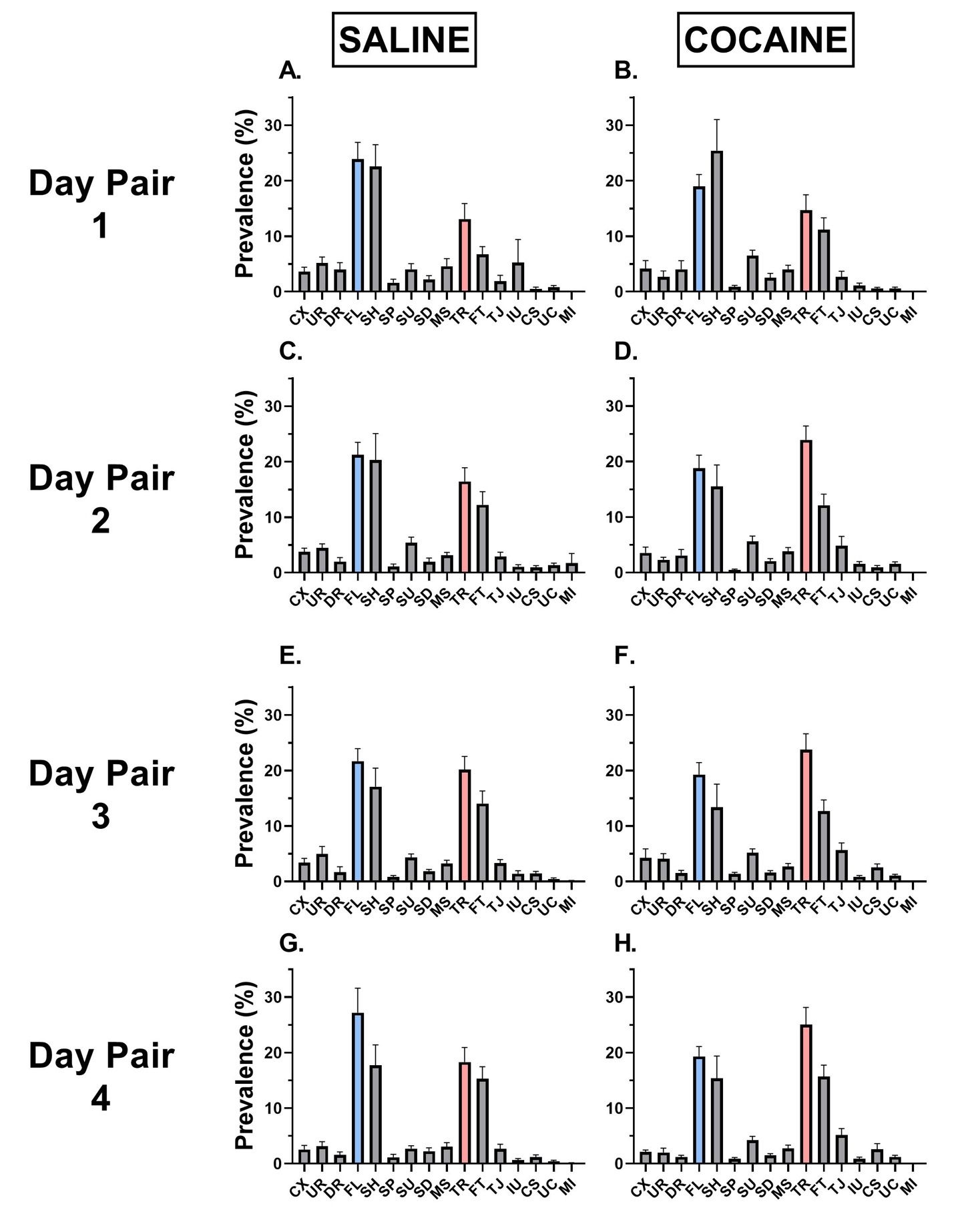


**Supplemental Figure 2. Call profiles across the conditioning sessions of Experiment 3** The y-axes show the percent prevalence of each call subtype (A-H). Call subtypes: CX complex, UR upward ramp, DR downward ramp, FL flat, SH short, SP split, SU step-up, SD step-down, MS multi-step, TR trill, FT flat-trill, TJ trill with jumps, IU inverted-U, CS composite, UC unclear, MI miscellaneous. The two pretreatment groups (i.e. saline and propranolol) were pooled, hence n=24 rats. Bars show the percent prevalence of each 50-kHz call subtype (mean + SEM) for the saline (left panels) and cocaine (right panels) conditions. The 8 conditioning days comprised 4 consecutive pairs of tests (i.e. Day Pairs). On a given Day Pair, each rat was tested once with saline and once with cocaine 10 mg/kg, in a counterbalanced order.
