# Supplementary material for: Adult rat ultrasonic vocalizations and reward: Effects of propranolol and repeated cocaine administration
Source: J Psychopharmacol. 2024 Aug 12;38(11):1025–41. doi: 10.1177/02698811241268894 (PMC11528876; doi:10.1177/02698811241268894)
Supplement: sj-docx-4-jop-10.1177_02698811241268894 – Supplemental material for Adult rat ultrasonic vocalizations and reward: Effects of propranolol and repeated cocaine administration [file sj-docx-4-jop-10.1177_02698811241268894.docx]

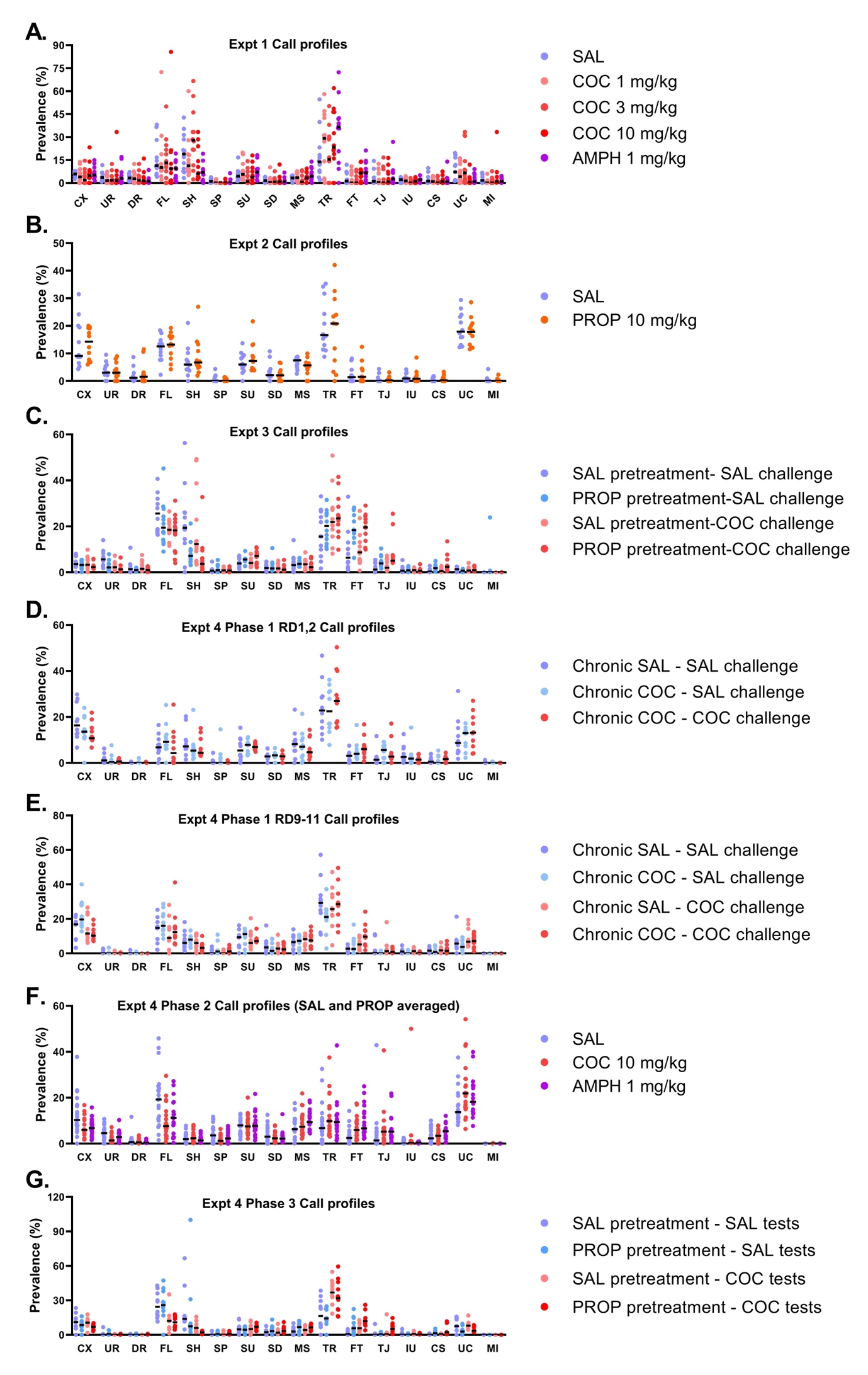


**Supplemental Figure 1.** **Call profiles for all experiments (Expt 1-3, and Expt 4 Phases 1-3)** The y-axes show the percent prevalence of each call subtype (A-G). Call subtypes: CX complex, UR upward ramp, DR downward ramp, FL flat, SH short, SP split, SU step-up, SD step-down, MS multi-step, TR trill, FT flat-trill, TJ trill with jumps, IU inverted-U, CS composite, UC unclear, MI miscellaneous. The n per drug condition for Experiments 1-4, was 12, 12, 24, and 20, respectively. Horizontal lines indicate the median value.
